# Establishment and application of a rapid new detection method for antimicrobial susceptibility testing of *Klebsiella pneumoniae* based on MALDI-TOF MS

**DOI:** 10.1128/spectrum.01346-24

**Published:** 2024-12-10

**Authors:** Yapei Zhang, Fanghua Fan, Xuan Wang, Jie Zhu, Shilei Dong

**Affiliations:** 1 Department of Clinical Laboratory, Zhejiang Hospital, Hangzhou, Zhejiang, China; NHLS Tygerberg/Stellenbosch University, Cape Town, Western Cape, South Africa

**Keywords:** *Klebsiella pneumoniae*, MIC, antibiotic susceptibility testing, MALDI-TOF MS, rapid method

## Abstract

The earlier appropriate treatment of *Klebsiella pneumoniae* infections according to the antimicrobial susceptibility profile based on the minimum inhibitory concentration (MIC) has a great clinical benefit. Our objective was to establish a matrix-assisted laser desorption/ionization time-of-flight mass spectrometry (MALDI-TOF MS)-based antimicrobial susceptibility test (AST) for *K. pneumoniae* that produces reliable results within a few hours. Our rapid method is similar to the classical turbidity-based microdilution method. We confirmed, theoretically and experimentally, that MALDI-TOF MS can replace the naked eye in judging the results (MIC_MS_) by “seeing” the bacterial growth in the presence of different concentrations of antibiotics, including determination of the lower limit of bacterial count detection (4 × 10^5^ cfu), the optimal period of incubation (2 h), and bacterial growth curve assay. Based on the study mentioned above, we determined the susceptibility of *K. pneumoniae* to imipenem. The MIC_MS_ and MIC data agreed over 85% (40/46) within 1 dilution range. Susceptibility profiles determined with our rapid method and the reference broth microdilution method were also compared. MIC_MS_ resulted in 97.9% (45/46) category agreement, 2.2% minor discrepancies, no major discrepancies, and no very major discrepancies. The summarized category agreement resulted in a kappa coefficient of almost 1 for weighted Cohen’s kappa, which could be considered a nearly perfect agreement. It took just 2 h to produce a susceptibility profile with a low failure rate using our new rapid AST method, a work day earlier than the broth microdilution method.

**IMPORTANCE:**

Empirical antimicrobial use before antimicrobial susceptibility test (AST) is necessary but risks patient harm and excess costs. It is particularly worrying that the inappropriate use of carbapenems has allowed carbapenem-resistant *Klebsiella pneumoniae* to become the commonest transmissible carbapenem-resistant Enterobacterales worldwide. Guidelines recommend targeted therapy based on minimum inhibitory concentration results, which directly reflect the effectiveness of antibacterial drugs. The gold standard method of AST relies on visible bacterial growth, causing long turnaround times. Current rapid AST techniques are hampered by factors such as high costs, technological complexities, and limited detection capabilities. We present a novel rapid method and applied to the determination of the susceptibility of *K. pneumoniae* to imipenem. It took just 2 h to produce a susceptibility profile with a low failure rate, a work day earlier than the standard method. Our method is potentially a faster, more precise, cost-efficient, and user-friendly AST method that can enhance the effectiveness of treatment strategies.

## INTRODUCTION

Although the empirical use of antimicrobial agents to treat infections before the antimicrobial susceptibility test (AST) is an inevitable and necessary measure to avoid a delay in treatment, the inappropriate use of antibiotics may be associated with patient harm, poor outcomes, and excess expenditure (1, 2). Clinical practice guidelines uniformly recommend the early conversion from an empirical to a targeted antimicrobial therapy based on the AST report released by a microbiology department. Previous clinical studies have indicated that the case fatality rate is significantly reduced among patients receiving concordant therapy relative to that of patients receiving discordant empirical antimicrobial therapy (3, 4). That is to say, the minimum inhibitory concentration (MIC) remains the standard criterion for the choice and administration of an antibiotic because it directly reflects the effectiveness of antibacterial drugs and assists clinicians to better match individual strains to the optimal antibiotic for their treatment (5).

Empirical antibiotic treatment based on clinical practice guidelines frequently recommends that broad-spectrum antimicrobial coverage should be broad enough to cover all potential pathogens (3). The clinicians’ decision threshold for the initiation of antibiotics in suspected infections was usually too low when patients had a high risk of mortality. Consequently, antimicrobials may be overused, undoubtedly escalating the frequency of the evolution of resistance during antibiotic exposure and contributing to bacterial resistance to these drugs (3). Of particular concern is the increased reliance on carbapenems as empirical agents, which has dramatically increased the numbers of carbapenem-resistant Enterobacterales (CRE) throughout the world. For most of the antibiotic era, the emergence of resistance generally follows closely upon the heels of the clinical introduction of antibiotics (6). Antibiotic resistance is an inevitable consequence of inappropriate antibiotic use.

Carbapenems, including imipenem and meropenem, are highly effective against Enterobacterales (including organisms resistant to the available third-generation and fourth-generation cephalosporins) and are typically considered drugs of last resort, reserved for patients with severe and complicated infections (7). The occurrence of CRE is an increasing public health threat worldwide. Accounting for the major proportion of CRE, carbapenem-resistant *Klebsiella pneumoniae* (CRKP) is especially worrying because it is often resistant to almost all categories of antibacterial agents currently available (8–10). Even more worrisome, the unnecessary or inappropriate use of carbapenems has allowed CRKP to become the commonest transmissible CRE worldwide according to several reviews that have assessed the evolution of CRE (11, 12).

The current gold standard method of AST is the broth microdilution method (BMM), a cumbersome and inefficient process, that requires at least 16–24 h to provide a final result. Although commonly used commercial automated microbiology systems are more maneuverable, they do not significantly reduce the turnaround time. Practically, a reduction in the total testing time may result in earlier appropriate treatment, ultimately reducing the morbidity and mortality associated with severe infection (3, 13). Previous research has focused on more rapid AST methods using polymerase chain reaction (PCR) or NG-Test CARBA 5 multiplex immunochromatographic assay (Fosun Diagnostics, China) for the detection of resistance genes or enzymes. However, the high cost of PCR and the lack of the necessary instrumentation and technical expertise in many diagnostic laboratories make it unsuitable for routine bacterial detection (5). Moreover, PCR detects only limited known genes or enzymes, so that organisms that express novel resistance mechanisms may go undetected. These two methods also do not reflect the real level of gene expression and enzymatic activity *in vivo* or the effectiveness of antibacterials. In conclusion, there is a critical need for a faster, more precise, cost-efficient, and user-friendly AST for effective anti-infective treatments (5).

The broth microdilution results cited above are interpreted based on the naked-eye observation of bacterial growth in the presence of antibiotics on the next day. If the drug has no inhibitory effect on the bacterium, the proliferation of the bacterium continues. However, the lead times on proliferation to the lower limit of growth that can be observed with the naked eye are at least 12–24 h, which is the primary cause of the long turnaround time for the final AST results.

Rapid species identification of the causative agents of microbial infections and the assessment of AST results are essential preconditions for prompt and appropriate antimicrobial therapy. The advent of matrix-assisted laser desorption/ionization time-of-flight mass spectrometry (MALDI-TOF MS) has accelerated bacterial identification and is widely used in clinical microbiology laboratories. The MALDI-TOF MS instruments that are used for microbial identification in the field are primarily supplied and supported by two companies, bioMérieux (France) and Bruker (Germany) (14). Research teams have preliminarily developed direct-on-target microdroplet growth assay for rapid AST (15–18), and these studies have been predominantly based on the MALDI-TOF MS system of Bruker, whereas there has been little preliminary testing of the MS apparatus from bioMérieux (Vitek MS) (18), which is commonly used in microbiology departments. Here, we present a newly developed method for the rapid detection of antibiotic resistance based on the reference BMM recommended by the Clinical and Laboratory Standards Institute (CLSI) guidelines for ASTs (19), and Vitek MS (bioMérieux, France) was used as a sharper “eye” instead of the unaided human eye to detect bacterial growth in the presence of different concentrations of antibiotics. Only about 2 h was required to report the MICs (MIC_MS_). We demonstrate the application of our method to the determination of the susceptibility of *Klebsiella pneumoniae* to imipenem, a classic example of carbapenems. The MIC_MS_ results obtained with this method were compared with those obtained with the methods currently used in our laboratory, hereafter referred to as “the current method”, i.e., the BMM or the disk diffusion method or an automated instrumental microbiology system based on Vitek 2 Compact (IM-VC; bioMérieux, France).

## MATERIALS AND METHODS

### Flow chart of the study outline

**Fig 1:**
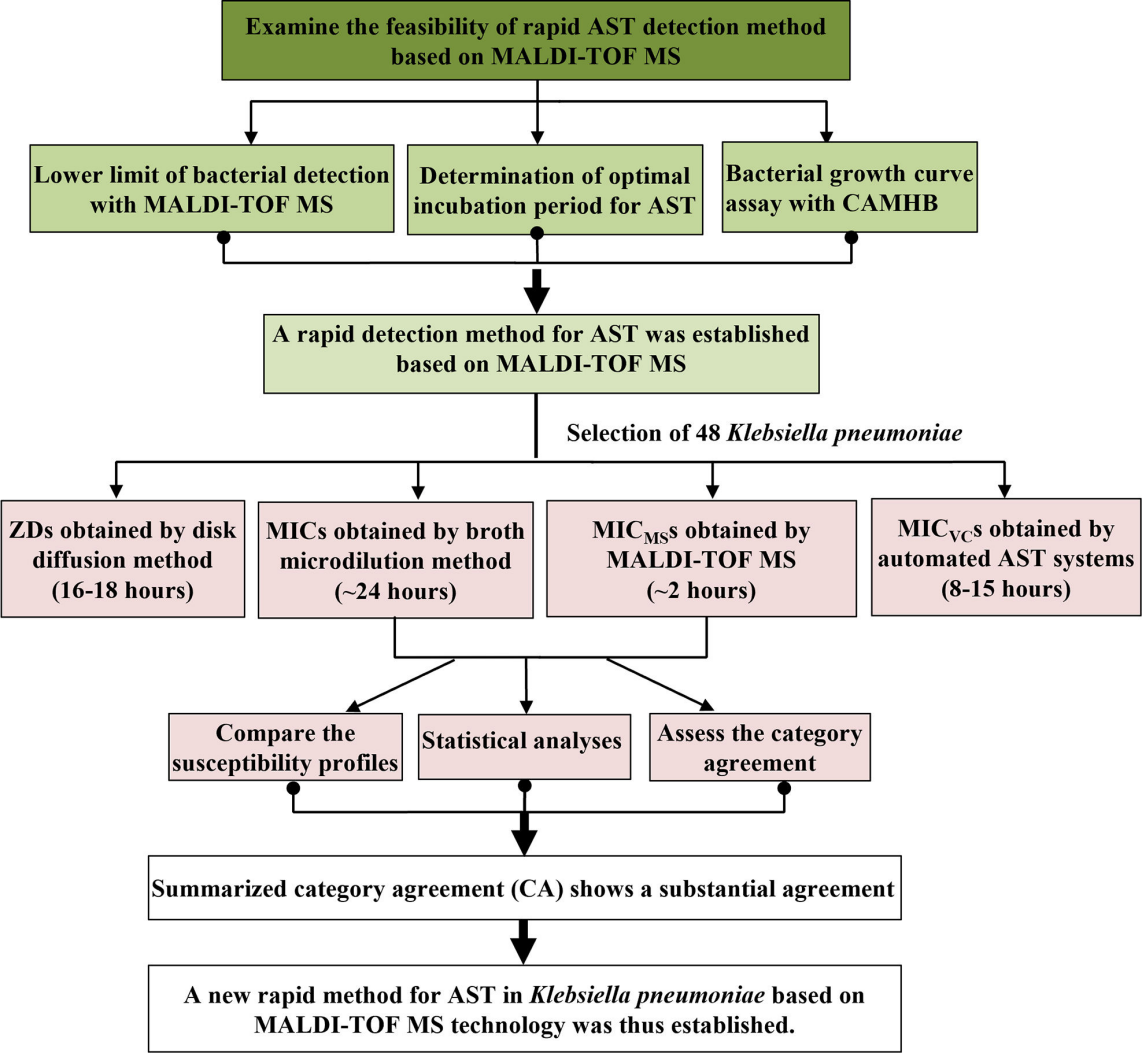
Flow chart outlining all the experiments and procedures included in this study. CAMHB, cation-adjusted Mueller–Hinton broth; ZDs, zone diameters; MIC_VC_s, the MIC values generated by IM-VC.

Figure 1 is a flow chart providing an overview of all the experiments and procedures included in this study. The study had three parts: the feasibility of the rapid detection method (RDM) based on MALDI-TOF MS (RDM-MS) was examined, the experimental procedures were formulated, and the method was applied to *K. pneumoniae*. The test results were summarized and the susceptibility profiles generated by BMM and RDM-MS were compared.

### Bacterial isolates

Forty-seven *K. pneumoniae* isolates were selected for analysis from 587 consecutive nonduplicate clinical *K. pneumoniae* isolates collected from all sites of inpatients at the Department of Clinical Laboratory, Zhejiang Hospital (Hangzhou, China), between August 2021 and July 2022. The same strain from a single patient was eliminated. Among which was 24 imipenem-susceptible and 22 imipenem-non-susceptible isolates randomly selected, and the only rough isolated in our laboratory selected at the beginning of the experiment. *Escherichia coli* ATCC 25922 was used for quality control for the MIC analyses. A total of 48 strains were examined. Initially, the bacteria were identified and their antimicrobial susceptibilities were established with the Vitek 2 Compact system (bioMérieux), and all the strains were further identified with the Vitek MS (bioMérieux).

The MIC range for imipenem was 0.125–256 µg/mL, covering the susceptible (MIC ≤ 1 µg/mL), intermediate (MIC = 2 µg/mL), and resistant categories (MIC ≥ 4 µg/mL) according to CLSI (19). The bacterial strains included all three types of bacterial colony morphology: smooth, mucoid, and rough.

### Lower limit of bacterial detection with MALDI-TOF MS instrument

During incubation, the initial inoculum size gradually increases to higher bacterial concentrations. Once the bacterial count is above the lower limit of detection with the MALDI-TOF MS instrument and allows the successful detection of bacterial colony-forming units (cfu), a sharp eye can see bacterial growth. Identifying this detection limit was the first step in developing our RDM-MS for AST. Briefly, the bacteria cultured overnight were adjusted to 0.5 McFarland standard (0.5 McFarland standard ~1.0 × 10^8^ cfu/mL) in sterile normal saline (NS) and diluted twofold with NS or cation-adjusted Mueller–Hinton broth (CAMHB) medium to different concentrations in the range of 80–1.25 × 10^5^ cfu/mL, in seven dilution steps. The number of bacteria in each dilution was determined by counting the colonies formed on Luria–Bertani (LB) agar plates, in duplicate samples. Then, 100 µL of each diluted bacterial suspension was added to a centrifuge tube (10 parallel samples of the same concentration were analyzed), and the cells were sedimented by centrifugation at 12,000 rpm for 10 min at room temperature. The bacterial counts corresponding to the seven dilutions mentioned above were 80 × 10^4^, 40 × 10^4^, 20 × 10^4^, 10 × 10^4^, 5 × 10^4^, 2.5 × 10^4^, and 1.25 × 10^4^ cfu, respectively. To avoid interference of the MALDI-TOF MS measurements by the ingredients of CAMHB (such as casein), the cells were washed with 100 µL of sterile NS, centrifuged, and the supernatant discarded. Because we detected nonspecific protein peaks in the blank control samples (aseptic CAMHB) in preliminary experiments, we used NS in washing steps to separate the microbial cells from CAMHB, as previously described (20). The samples mixed with NS did not require this step. The bacterial cells were suspended in 1 µL of Vitek MS-CHCA matrix and transferred to a Vitek MS target plate (bioMerieux). MALDI-TOF MS was performed routinely to acquire the spectra on the Vitek MS instrument calibrated with a standardized control, *Escherichia coli* ATCC 8739. All samples were assayed with routine U.S. Food and Drug Administration-approved *in vitro* diagnostics (IVD), using the standard manufacturer-recommended settings. A score of ≥90% based on the standard clinical Vitek MS software confidence levels intended for organism identification was deemed a success. Conversely, nonidentification on Vitek MS was interpreted as an unsuccessful result. The experiment was repeated independently in triplicate. The lowest diluted bacterial suspension at which all 30 samples were successfully identified (score ≥ 90%) was determined as the lower limit of detection.

### Determination of optimal incubation period for RDM-MS

The optimal incubation period to detect bacterial growth is the shortest period required to confirm that the bacterial count has increased from the initial inoculum size to the lower limit of detection of the MALDI-TOF MS instrument. We randomly selected one clinical strain and prepared 21 tubes containing 100 µL of CAMHB medium with the bacterium (200-fold dilution of the 0.5 McFarland standard). Three samples were centrifuged, washed, and analyzed with MALDI-TOF MS as described above. The other 18 tubes were placed in incubators, and three samples were removed every hour for 6 h to observe the bacterial growth with MALDI-TOF MS. We selected the time point at which all three samples were successfully identified as the optimal incubation period for RDM-MS. The experiment was independently repeated in triplicate.

### Bacterial growth curve assay

**Fig 2:**
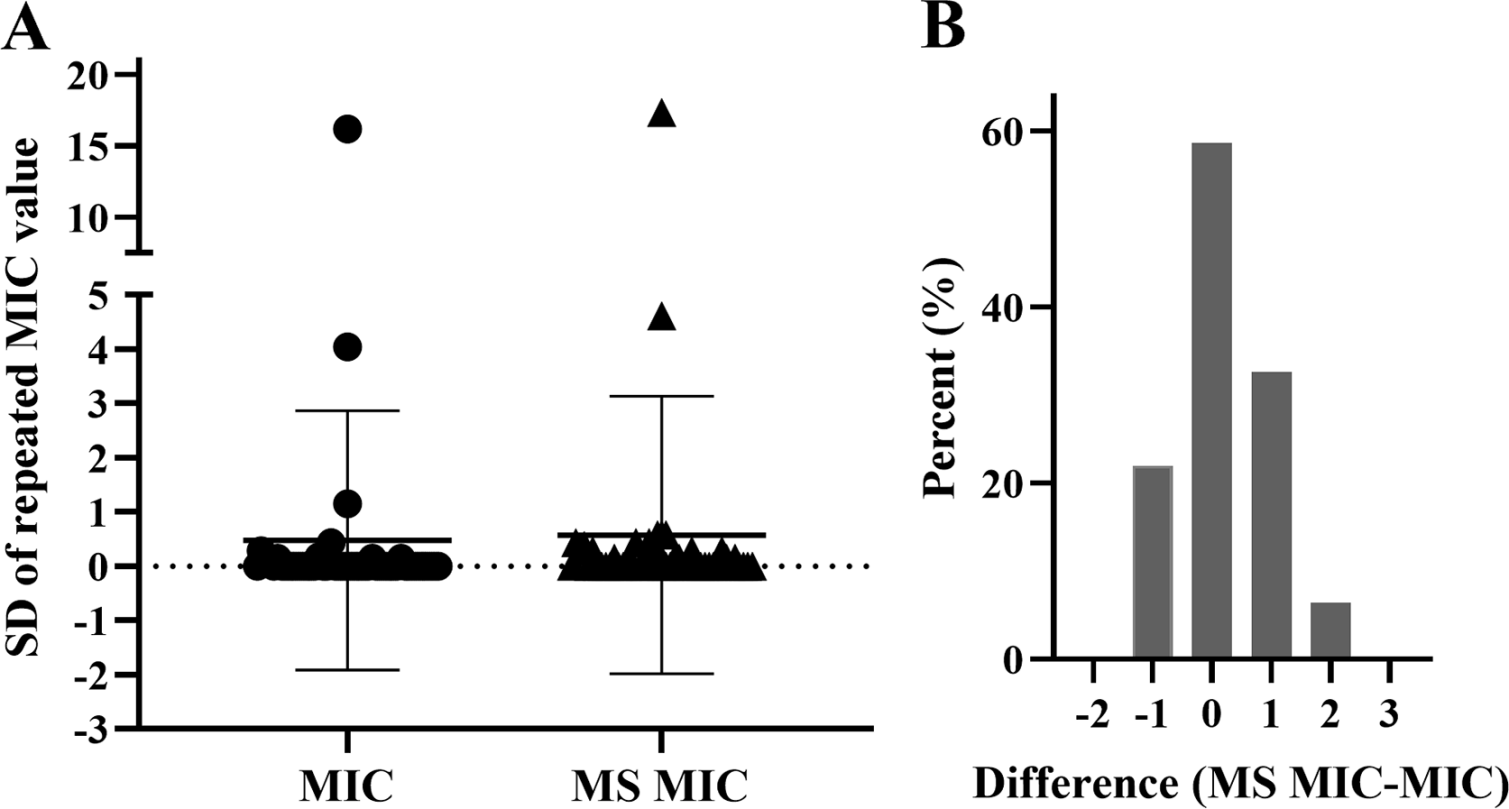
(**A**) Comparison of the reproducibility of MIC_MS_ and MIC. Relative units on the *y*-axis represent the standard deviation (SD) of three independent replicates. (**B**) Distribution of MIC differences in the two tested methods, where “1” represents a twofold dilution difference. Negative values represent MIC_MS_s lower than MICs, positive values represent MIC_MS_s values higher than MICs, *n* = 46, two invalid data were excluded.

To confirm the reliability of the conclusion drawn from the experiment described above, a bacterial growth curve was constructed as previously described (21) with minor modifications. In this experiment, LB broth, a commonly used microbial culture medium, was replaced with CAMHB, which is recommended by the CLSI guidelines as the best nutrient medium for the gold standard AST method. The colony type of the *K. pneumoniae* isolates was either smooth, mucoid, or rough (Fig. 2). We randomly selected one isolate from each different colony type to construct the growth curves. Briefly, the selected isolate was grown in static culture at 37°C, in tubes containing 100 µL of fresh CAMHB medium with the bacterium (200-fold dilution of 0.5 McFarland standard). One sample was taken every 30 min or every 1 h for 7 h, and the number of bacteria was determined at each time point by counting the colonies on LB agar plates in triplicate. The mean number of bacteria was calculated for further analysis. The experiment was repeated independently in triplicate. The growth curves were plotted, with the number of bacteria (log10 cfu/mL) as the ordinate and time (h) as the abscissa.

### AST of bacteria with RDM-MS

Tubes containing antibiotics were prepared and inoculated with bacteria as for the broth microdilution reference method (the gold standard AST method), according to the latest CLSI guidelines (19). *Escherichia coli* ATCC 25922 was used for quality control, and its MIC was within the quality control ranges throughout the study. In brief, a 0.5 McFarland bacterial suspension was diluted 1:200 in CAMHB. The final concentration of the bacteria used for inoculation was standardized to approximately 5 × 10^5^ cfu/mL. Then, 100 µL of the diluted bacterial suspension was added to tubes preloaded with serially diluted lyophilized imipenem (Kangtai, Wenzhou, China), which was dissolved to 0.125–256 µg/mL in the appropriate solvent. The number of colony-forming units in the initial inoculum was approximately 5 × 10^4^ cfu, which was confirmed with colony counts of serial dilutions in CAMHB. Parallel wells containing the bacterial suspension without antibiotics were included as growth controls. Parallel wells containing sterile broth were included as blank controls. The tubes with bacteria and antibiotics were sealed and incubated at 37°C under aerophilic conditions. After 2 h, all samples were centrifuged and washed, and MALDI-TOF MS detection was performed, as in the previous steps. MIC_MS_ was read as the lowest antibiotic concentration at which no identification was achieved with the Vitek MS software. Samples with concentration of antibiotics below the MIC_MS_ should show successful identification (score ≥ 90%). The test was deemed valid if the measurement of a sample lacking antibiotic (growth control) resulted in successful species identification (score ≥ 90%). The experiment was independently repeated in triplicate, and the median MIC_MS_s were calculated for further analysis.

### AST of bacteria with the current reference method

Tubes with bacteria and antibiotics were prepared and inoculated as in the broth microdilution reference method, which is the current reference method. The MICs were analyzed after 24 h (due to daily work schedule issues) and were read as the lowest antibiotic concentration resulting in the complete inhibition of visible growth. Each experiment was repeated independently three times, and the median MICs were calculated for analysis. These were used as the comparators for the evaluation of the MIC_MS_ results obtained with MALDI-TOF MS.

The susceptibility of the isolates to imipenem was also determined with the disk diffusion method according to the latest CLSI guidelines (19).

### Summary of testing results and statistical analysis

We summarized the test results, compared the data point by point, and compared the susceptibility profiles generated with microdilution and MALDI-TOF MS according to CLSI guideline M52 (21), including category agreement (CA), essential agreement (EA), major error (ME), and very major error (VME). AST methods are considered verified for a specific drug/microorganism combination when CA and EA are ≥90%, the VME rate is <3% of the total resistant isolates, the ME rate is <3% of the total susceptible isolates (22). We also compared the MIC values generated by the two methods and drew a heatmap to show visually the differences in the data and the consistency of the data.

The statistical analyses and the comparison of MIC values were performed with GraphPad Prism 9 (Boston, MA, USA). Differences were deemed significant at *P* < 0.05. Weighted Cohen’s kappa calculated with Medcalc (version 20.0.22) (Acacialaan, Ostend, Belgium) was used to assess CA between MIC_MS_ and MIC.

## RESULTS

### Lower limit of bacterial detection with MALDI-TOF MS instrument

Therefore, we detected different lower limits of bacterial detection when the nutrient medium was CAMHB or NS. All 30 samples were successfully identified (score ≥ 90%), and those containing CAMHB medium had the same bacterial count of 4 × 10^5^ cfu, whereas samples containing NS medium had a count of 5 × 10^4^ cfu (Table 1).

**TABLE 1:** Lower limit of bacterial detection with the MALDI-TOF MS instrument^*a*^

Bacterial count (×10^4^ cfu)	1.25	2.5	5	10	20	40	80
Normal saline	0	0	**100%**	100%	100%	100%	100%
CAMHB	0	0	0	0	16.7%	**100%**	100%

^*a*^ Percentages represent the ratio of the successfully identified samples to all the samples in each category. The values in bold indicate the lower limits of bacterial detection with the MALDI-TOF MS instrument, which showed the best assay performance.

### Incubation time for reliably detectable bacterial growth

When developing rapid methods of ASTs, the initial bacterial concentration affects the assay results (18). Therefore, to establish a new rapid method entirely based on the broth microdilution reference method, we chose a bacterial concentration of 0.5 McFarland standard diluted 200-fold as the initial inoculum in this study. Preliminary experiments showed that an incubation period of 2 h was insufficient to achieve consistent results for *K. pneumoniae*, mainly because the growth controls did not reach the Bruker MS detection limit (15) in that time, but was sufficient to reach the Vitek MS detection limit (18). Because previous studies have reported different incubation times when developing different rapid AST methods (15, 18), we tested incubation times ranging from 1 to 6 h to determine the optimal incubation time for reliably detectable bacterial growth, while still allowing same-day MIC results. After incubation for 2 h, valid identification results were achieved with MALDI-TOF MS for all isolates. We also determined the number of bacteria (range: 4.3–9.3 × 10^5^ cfu) per 100 µL at each time point, and this was always definitely >4 × 10^5^ cfu, the lower limit of bacterial detection of the MALDI-TOF MS instrument. Therefore, in subsequent experiments, we used 2 h incubation for all MIC_MS_ measurements.

### Bacterial growth curve assay of *K. pneumoniae* in CAMHB

**Fig 3:**
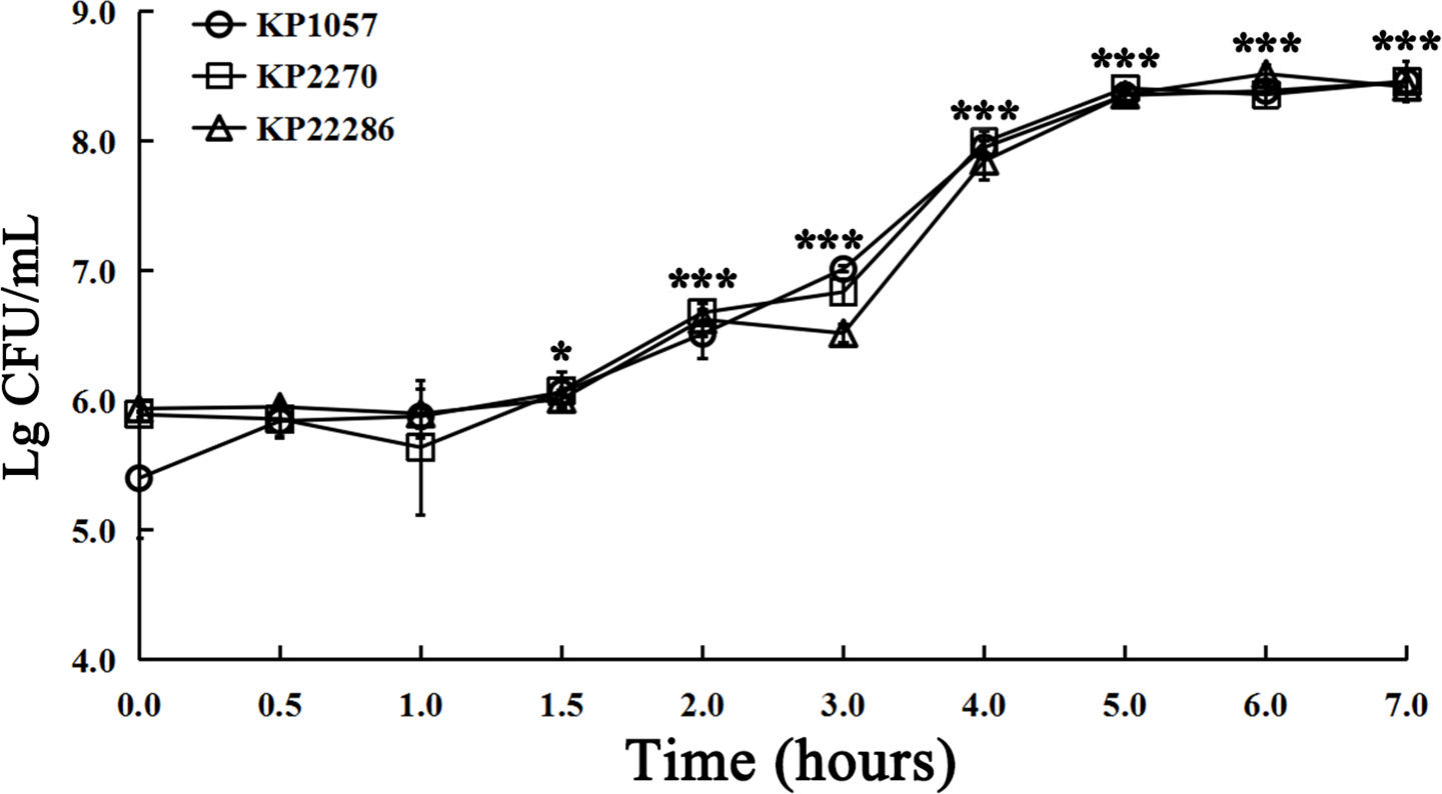
Growth curve of three isolates with different colony types. KP1057, KP2270, and KP22286 represent the smooth type, mucoid type, or rough type, respectively. Refer to Fig. 4 for the three bacterial colony morphologies. *P < 0.05.

To more directly describe the growth kinetics of bacteria in a nutrient medium for the gold standard AST method, we drew the growth curves of *K. pneumoniae* in CAMHB, rather than in other common microbial culture media. The growth curve showed the lag phase of growth occurred at about 1–1.5 h, and the growth rate was low. The bacteria reached the log phase and then grew exponentially (Fig. 3). After incubation for 2 h, the bacterial count increased continuously in all the bacteria tested, and the numbers of bacteria were potentially detectable with the MALDI-TOF MS instrument.

**Fig 4:**
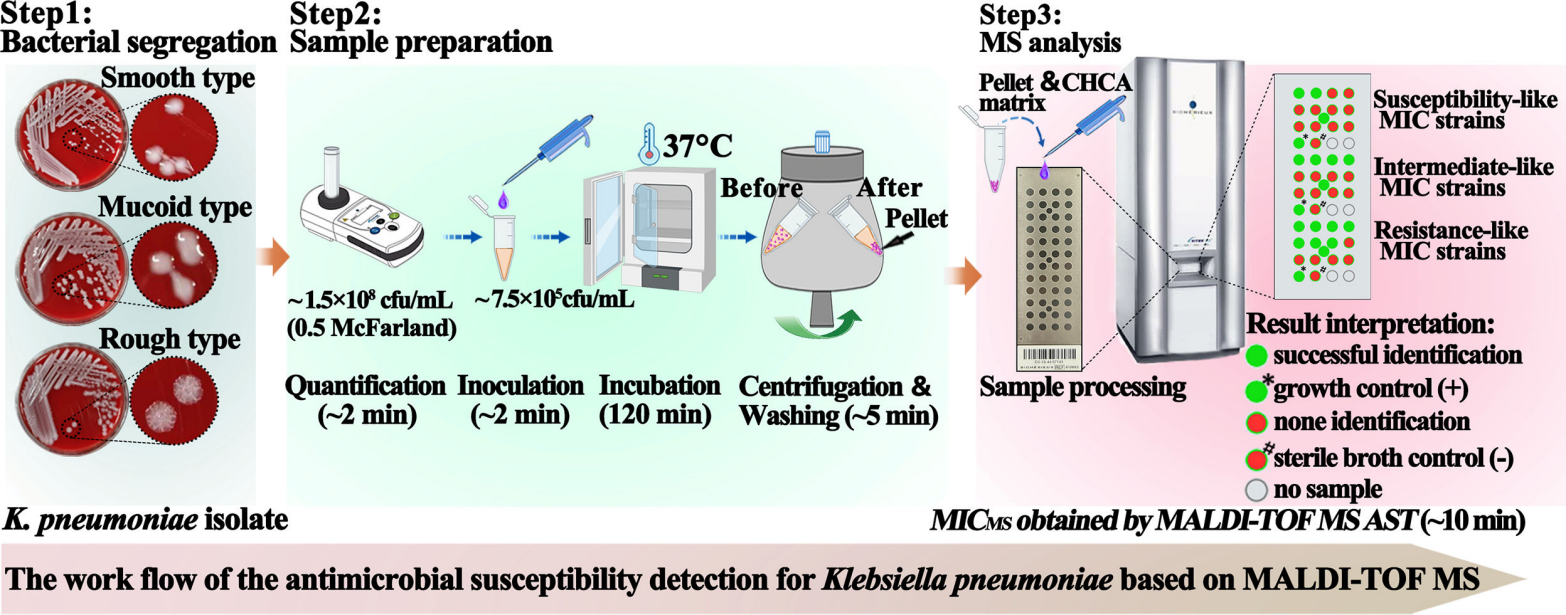
Experimental workflow of the rapid detection method based on MALDI-TOF MS.

### Summary of test results

**Fig 5:**
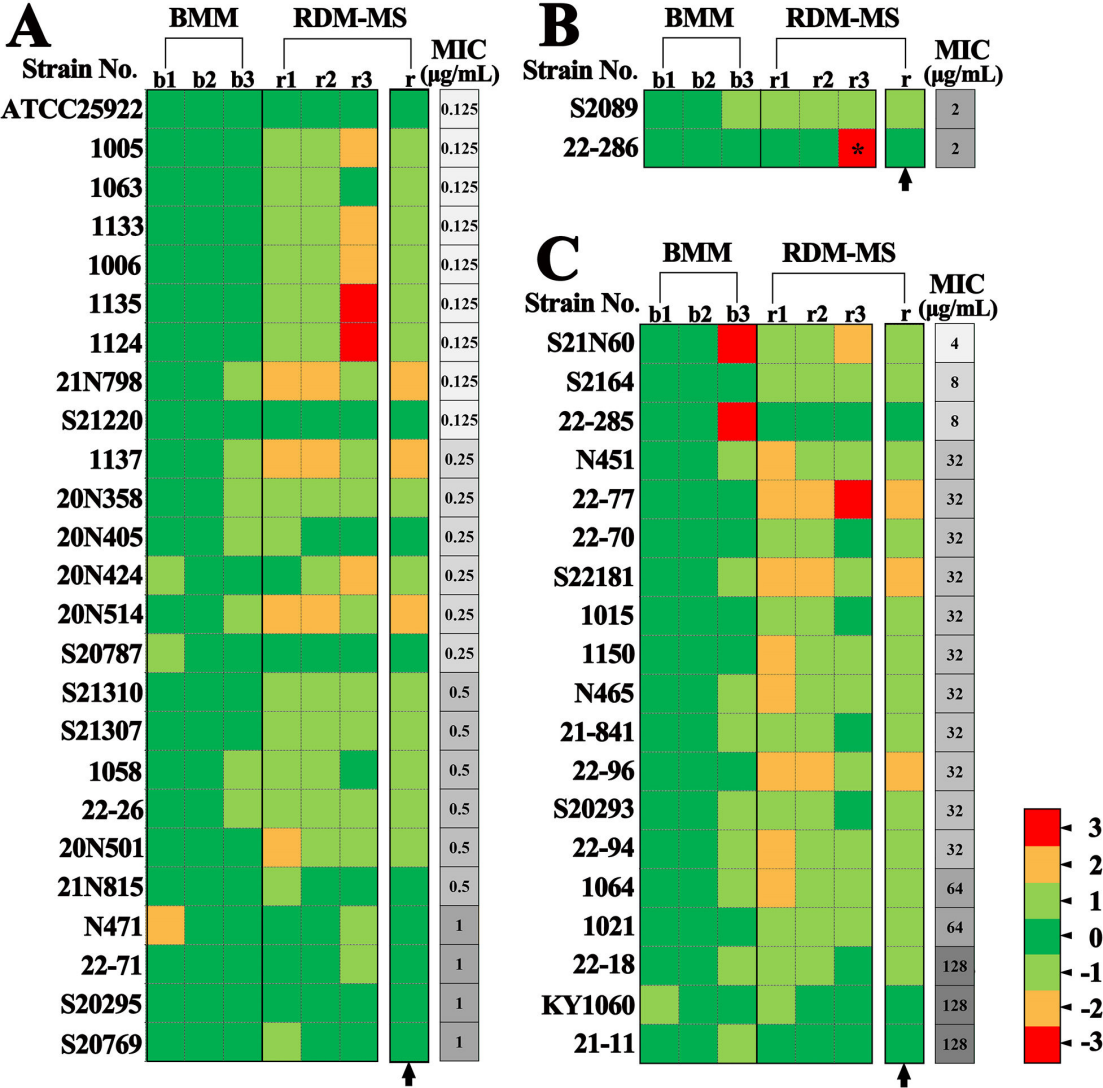
Comparison of MIC values generated by BMM and RDM-MS. (**A**) Susceptible MIC strains. (**B**) Intermediate MIC strains. (**C**) Resistant MIC strains. Bacterial classification according to the MICs obtained with the broth microdilution reference method (the gold standard AST method). Arrow indicates MIC_MS_s measured with RDM-MS. “1” represents one doubling dilution step difference, “2” represents two doubling dilution step difference, “3” represents three doubling dilution step difference, * represents more than three doubling dilution step difference. Negative values represent MIC_MS_s lower than MICs, positive values represent MIC_MS_s values higher than MICs.

We tabulated the MIC_MS_ values generated by RDM-MS and the MIC values generated by BMM (Table S1). In our rapid AST, CA was 97.9% (45/46), and EA (MIC pairs matched within ±1 dilution range) was 87.0% (40/46). Both ME and VME were 0%. The agreement between MIC_MS_ and MIC was good, which was also illustrated by the heatmap about the comparison of MIC values generated by the two methods (Fig. 5). The invalid data for two mucoid-type isolates, which had hypermucoviscous phenotypes and generated >30 mm viscous strings, were excluded from the analysis (Table S1).

We also compiled the MIC values generated with IM-VC (Table S1) and drew a heatmap (Fig. S1), which showed the poor consistency between RDM-MS and IM-VC.

Because Vitek’s maximum dilution for imipenem is ≥16 µg/mL, IM-VC could not report specific values, as RDM-MS and BMM did. This was not the main scope of our study, and we undertook no further detailed analysis.

The inhibition zone diameters induced with imipenem are shown in Table S1. The relevant content will be covered further below when we discuss the reliability of our new method in the following discussion.

### Comparison of MIC values

First, we compared the standard deviation (SD) of MIC_MS_ and MIC. The pattern of the SD and the average SD of MIC_MS_ were similar to those of the reference method (Fig. 2A), suggesting that the reproducibility of MIC_MS_ is similar to that of the microdilution method. Intriguingly, two data points deviated significantly between the two sets of data, which were associated with heteroresistant strains.

We then compared the distribution of MIC differences between the two tested methods. MIC_MS_ values were slightly higher than the MICs from microdilution (Fig. 2B). We then calculated the bias in the MIC_MS_ measurements according to the ISO/DIS 20776-2 standard. The percentage of MIC_MS_ values greater than the MICs of the reference method was 39.1% (18 of 46 measurements), and the percentage of MIC_MS_ values less than the reference MIC was 2.2%; therefore, the calculated bias was 36.9%.

Finally, we determined weighted Cohen’s kappa to further analyze CA (Table 2). Cohen’s weighted kappa coefficient was 0.978 (95% CI: 0.935–1.000), indicating that the two methods were highly consistent and showed substantial interobserver agreement.

**TABLE 2:** Comparison of susceptibility profiles defined by MIC or MIC_MS_*^a^*

MIC_MS_	MIC			Cohen’s weighted kappa
	S	I	R	
S	25	0	0	0.978 (95% CI: 0.935–1.000)
I	0	1	0	
R	0	1	19	

^*a*^ R, resistance; I, intermediate; S, susceptibility.

## DISCUSSION

**Fig 6:**
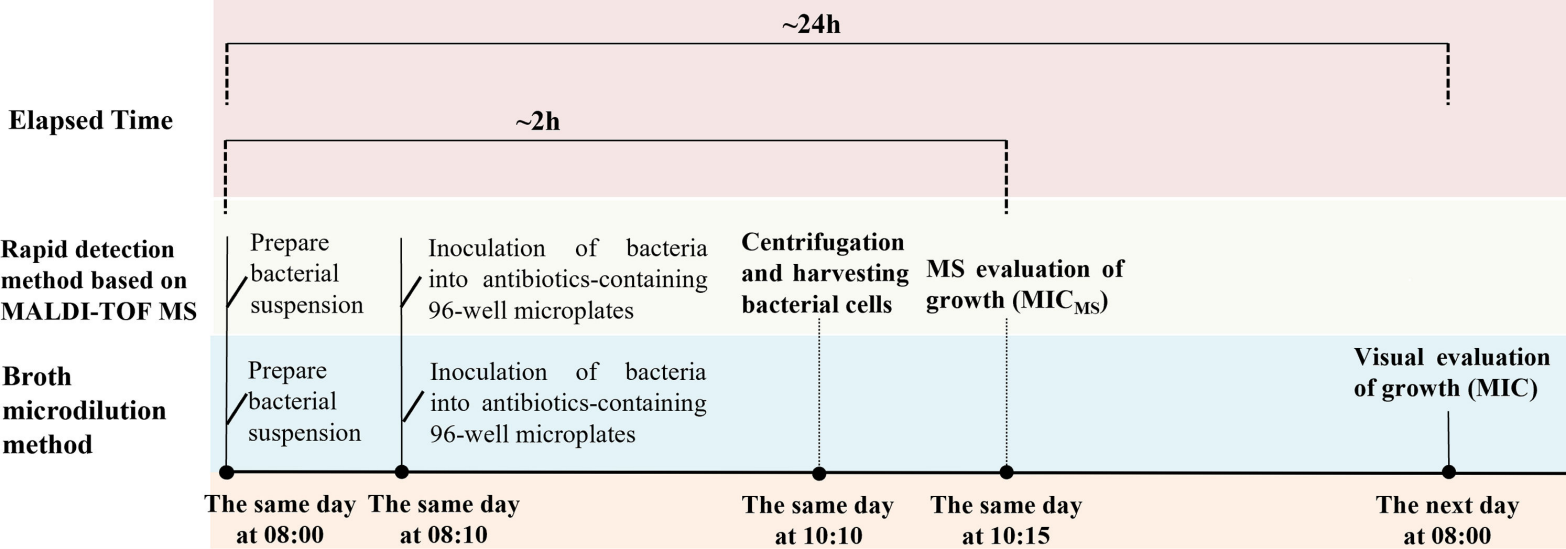
Experimental workflow of BMM and RDM-MS.

Microbiology departments usually report MICs on the day after test initiation with the gold standard broth microdilution method or common commercial automated microbiology systems. In an emergency, empirical antibacterials, including carbapenems, are generally used to avoid any delay in the treatment of infections. However, targeted antibiotic treatments based on MICs are much more effective (23, 24) and may improve clinical outcomes, reduce mortality and the length of stay, and even minimize the evolution of resistance to carbapenems (24). Reducing the AST turnaround time can accelerate clinical decision-making about personalized medicine. Up-to-date clinical guidelines have identified rapid AST results as one of the issues most urgently requiring improvement, and numerous studies have reported methods that can determine the susceptibility profile of a pathogen faster than the standard method (25). However, no test has proved superior to the classical microdilution method in clinical practice. In this study, we developed a new RDM for the AST of imipenem in *K. pneumoniae*, similarly based on the gold standard broth microdilution method but using the MALDI-TOF MS technology. The marked difference between the two methods is the length of the AST, which we reduced by reducing the incubation time to 2 h, to deliver same-day MIC results (Fig. 4 and Fig. 6). The RDM-MS is as reliable as the gold standard method, and in some cases, is more accurate than the most commonly used automated AST system.

RDM-MS is similar to the turbidity-based classical microdilution method. Tubes containing antibiotics are prepared and inoculated with bacteria, similarly to BMM, and then incubated at 37°C under aerobic conditions. Finally, all the samples are subjected to MALDI-TOF MS detection to determine the bacterial growth in the presence of different concentrations of antibiotics (Fig. 4). The lower limit of bacterial detection with the MALDI-TOF MS instrument reflects how sharp the new “eye” is in our RDM for the AST. The most critical and first step in RDM-MS is to determine how many bacterial cells will be “seen” by MALDI-TOF MS and “judged” to be muddy. Nonspecific protein peaks were detected in the blank controls (aseptic CAMHB) with MALDI-TOF MS in the preliminary experiments, indicating that the components of the nutrient broth that are required for a bacterium’s growth may disturb MALDI-TOF MS measurements. Casein is one of the important components of CAMHB and may be the main factor interfering with MALDI-TOF MS measurements used to establish bacterial protein profiles. It has been reported previously that the culture medium may influence the efficiency of bacterial identification (14). To analyze liquid cultures, prepurification is mandatory, and the most popular method combines centrifugation and washing steps (14). CAMHB is specifically recommended by the CLSI guidelines as the nutrient medium for the gold standard AST method. Therefore, we used prepurification to limit the interference caused by components of CAMHB. The lower limit of bacterial detection with NS was 5 × 10^4^ cfu, which is consistent with the amount of microbial biomass (10^4^–10^5^ cfu) that can be identified with MALDI-TOF MS (26). Therefore, NS did not disturb MALDI-TOF MS measurements, scientifically supporting the use of NS in the washing steps in subsequent experiments. The lower limit of bacterial detection with CAMHB was 4 × 10^5^ cfu, which is about an order of magnitude higher than 5 × 10^4^ cfu, the initial inoculum size recommended by the CLSI guidelines for the AST, but is not detectable with MALDI-TOF MS. During incubation, higher bacterial concentrations were successfully detected when they exceeded the lower limit of detection by MS. The lower limit of bacterial detection played a key role in the development of our RDM-MS and validates the use of ASTs based on the MALDI-TOF MS technology.

Reducing the period required for the AST has many benefits, but an inadequate incubation time can result in misleading susceptibility profiles, and thus lead to inappropriate clinical decisions. With a rigorous study, we confirmed that the optimal incubation time for reliably detectable bacterial growth is 2 h, which is 1 h shorter than the incubation duration reported in previous studies that sought to rapidly determine the susceptibility profiles of pathogens (15, 20). In terms of the specific experimental process, previous studies were based on a direct-on-target microdroplet growth assay of 2–10 µL of bacterial suspension (200-fold dilution of 0.5 McFarland standard), but 100 µL of bacterial suspensions at the same concentration was used to prepare the bacterial cell pellets in the present study. In other words, when the volume of suspension was increased, the bacterial counts exceeded the detection limit of the MALDI-TOF MS instrument in a shorter period. In contrast, previous studies acquired spectra with the instrument settings used for routine bacterial identification, with only slight modifications, including the addition of shots and changes in the movement raster (15). Our assay relies entirely on the Vitek MS IVD software, without modification, which offers simple implementation for routine use. Furthermore, our incubation time is virtually the same as that in a liquid extraction method that predicts antibiotic resistance based on the Vitek MS instrument (18). Although the original volume of the suspension is not given in detail, we speculate that at least 100 µL or even more was used according to the established reference method (18, 27). Furthermore, the number of bacteria was in the range of 4.3–9.3 × 10^5^ cfu after incubation for 2 h, which is well above the lower limit of bacterial detection with MS, 4 × 10^5^ cfu.

The results of the practical work described above confirm that 2 h for the AST is completely feasible. From our theoretical analysis, for good-quality phenotypic AST results, the incubation time should be long enough to allow logarithmic bacterial growth because bacteria showed no clear growth to the limit of detection during the lag phase, which usually lasts about 2–5 h for different organisms (15, 18). In our study, the bacterial growth curve for *K. pneumoniae* grown in CAMHB provided the scientific basis for choosing 2 h as the optimal incubation time and confirmed the reliability of the practical process. According to the theoretical analysis and its practical verification, this approach is completely feasible. Our theoretical analysis and experiments were well matched. Based on the research results described above, selecting 2 h as the optimal incubation time is not only a practical conclusion but is supported by convincing scientific evidence.

The currently available AST methods usually require up to 24 h to provide results, given the daily workflow. The experimental setup for the MS AST is based on the microdilution method (Fig. 4), and the MIC results can be read a workday earlier than BMM (Fig. 6).

Of all the elements of feasibility, quality performance is more critical than time. Therefore, we compared the reproducibility and reliability of MIC_MS_ with those of the gold standard method.

We undertook a preliminary analysis of the MIC_MS_ values generated by RDM-MS and the MIC values generated by BMM (Table S1). No valid MIC_MS_ of two mucoid-type isolates could be generated with MALDI-TOF MS, and these samples were excluded from the analysis. According to the MALDI-TOF MS instructions, further processing of these mucoid bacteria, which produce an exopolysaccharide matrix, is required to produce good-quality mass spectra. Each colony is overlain by 70% formic acid, and after drying, by the MS-CHCA matrix. After many attempts, the measurement of growth control still did not successfully identify the species, so the test was deemed invalid and the samples were excluded. This failure is related to the working principle of the MALDI-TOF MS: protein mass spectra that are mainly based on ribosomal proteins are used to group and identify bacteria (26). The slime of the mucoid bacteria is mainly composed of exopolysaccharide (28), which forms a complex mass that is difficult to accurately resolve and is consequently a major source of interference. Our new RDM is based on MALDI-TOF MS and therefore is limited by the limitations of MALDI-TOF MS. Therefore, it is not always applicable to mucoid-type bacteria, especially hypermucoviscous bacteria, which form strings of >30 mm (Table S1).

According to the SD of the replicates, the reproducibility of MIC_MS_ did not differ significantly from that of MIC (Fig. 2A). All the data points clustered closely around the mean values, except for the two points related to two strains that were heteroresistant to imipenem. These appeared as discrete colonies with clear zones of inhibition after incubation in disc diffusion assays (data not shown). One of the two strains formed rough-type colonies. For this strain, the automated microbiology system occasionally indicated sensitivity to imipenem, whereas both broth culture and MS showed it to be drug-resistant (Table S1). Repeated testing with the disk diffusion method confirmed its drug resistance. Therefore, our RDM-MS provides both specific numerical values similar to the reference method, but with more accurate results than the automated microbiology system. A limitation of our study was that it included a limited number of heteroresistant and/or rough strains, mainly because the rate at which these strains are isolated at our medical center is low.

Although the criteria are not fully met by our rapid AST system (EA = 87%) according to CLSI guideline M52, the system has been preliminarily verified, and our achievement is a promising basis for further studies to refine the methodology to meet these criteria. Besides, the bias of the MIC_MS_ values was 36.9%, which was probably related to the nonuniform distribution of bacterial strains among different strain types. For example, the numbers of rough strains and intermediate MIC strains were small (Table S1).

To sum up the discussion, the above discrepancies, including the SD of the replicates, the bias, and the 87% EA (falling short of the 90% EA target), mainly arose from the presence of relatively infrequent strain types in the samples. In clinical practice, heteroresistant strains, rough strains, and intermediate MIC strains are isolated less often than smooth and mucoid strains. For more comprehensive verification, these infrequent strains should be collected over a longer period. On the positive side, these discrepancies do not alter the susceptibility profiles according to the CLSI breakpoints (Table S1). The summarized CA resulted in a kappa coefficient almost equal to 1 for weighted Cohen’s kappa (Table 2), showing substantial interobserver agreement, which could be considered an almost perfect agreement. Moreover, our RDM is based on microbiological experience, so there may be no need to generate new clinical standards, and the defined phenotypic MIC_MS_ values can be interpreted according to the clinical breakpoint standards of CLSI. Although our results do not fully meet the rigorous criteria for AST, MIC_MS_ is a promising basis for further investigations to refine the methodology to meet these criteria.

In addition to the time and quality performance of our RDM-MS, other aspects of the AST were investigated. To increase the ease of sample processing is another impetus behind the evolution of AST methods. MS is a robust technique and excels in high-throughput measurements. Vitek MS calculates the MIC value of imipenem for a single isolate at multiple concentrations in several minutes, which makes it possible to automate the test. High-throughput and automatized processing raise the determination of MIC_MS_ to the standard of an ideal AST.

The MIC values of our tested strains covered a wide and comprehensive range, including susceptible, intermediate, and resistant categories, which compensated for the lack of intermediate MIC strains in previous research (18). To the best of our knowledge, this study is the first to use Vitek MS for the rapid detection of bacterial antibiotic susceptibility simultaneously in bacteria with all three types of bacterial colony morphology (smooth, mucoid, and rough). We have also described the effect of adding formic acid on the experimental results, although no relevant descriptions were given in other studies of rapid MIC detection (15, 16, 18, 29). All these extensive validations are essential for the application of our new RDM to the AST in routine clinical laboratory practice.

Our RDM-MS reduces the overall turnaround time for the MIC results of imipenem in *K. pneumoniae* and is certainly useful to guide for the empirical use of imipenem in clinical practice and to assist physicians whether to select imipenem as anti-infective agent for individual patients. Using the imipenem reasonably may also help to remove the selective pressure on pathogens and reduce the overall exposure to imipenem, which promotes the emergence of carbapenem resistance mechanisms.

### Conclusion

We have comprehensively described a new rapid method for the AST of imipenem in *K. pneumoniae* based on the MALDI-TOF MS technology, which may be suitable for routine microbiological laboratory practice and has great diagnostic potential. Unlike other works covering a wider range of species and antibiotics, our work only tests against one antibiotic and one species, but our tested strains covered a wide MIC range and comprehensive colonial morphology. To identify the real advantages and clinical applicability of MIC_MS_, further testing is required on more clinical samples, especially heteroresistant and intermediate-MIC strains and/or rough strains. This will clarify the efficacy of our method in predicting the susceptibility of *K. pneumoniae* isolates from other laboratories and even other countries.

## SUPPLEMENTAL MATERIAL

Fig. S1 spectrum.01346-24-s0001.docx Comparison of the MIC values generated with IMVC or RDM-MS.

Table S1 spectrum.01346-24-s0002.xlsx Experimental data.

ASM does not own the copyrights to Supplemental Material that may be linked to, or accessed through, an article. The authors have granted ASM a non-exclusive, world-wide license to publish the Supplemental Material files. Please contact the corresponding author directly for reuse.
