# Supplementary material for: Establishment and application of a rapid new detection method for antimicrobial susceptibility testing of Klebsiella pneumoniae based on MALDI-TOF MS
Source: Microbiol Spectr. 2024 Dec 10;13(1):e01346-24. doi: 10.1128/spectrum.01346-24 (PMC11705933; doi:10.1128/spectrum.01346-24)
Supplement: Fig. S1 — Comparison of the MIC values generated with IMVC or RDM-MS. [file spectrum.01346-24-s0001.docx]

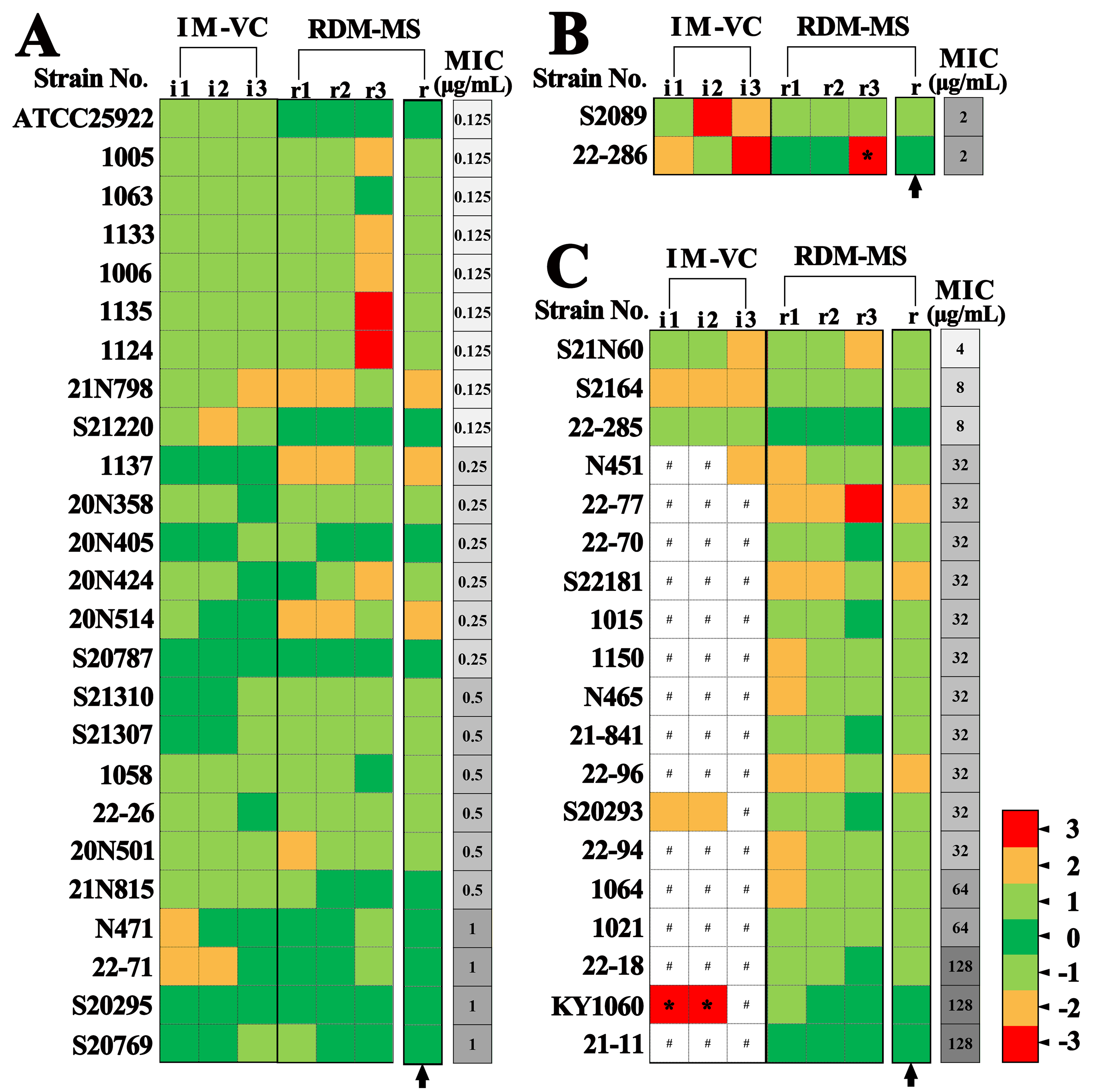


**Fig S1.** Comparison of the MIC values generated with IMVC or RDM-MS. (A) Susceptible strains. (B) Intermediate strains. (C) Resistant strains. Bacterial classification according to MIC was achieved with the broth microdilution reference method (the gold standard AST method). Arrows indicate the MICMS measured with RDM-MS. “1” represents one doubling dilution step difference, “2” represents two doubling dilution step difference, “3” represents three doubling dilution step difference, * represents > three doubling dilution step difference; # represents MIC values exceeding the detection limit of IMVC (16 μg/mL). Negative values represent MICMSs lower than MICs; positive values represent MICMSs values higher than MICs.
